# LB4. Casirivimab and Imdevimab for Treatment of Hospitalized Patients With COVID-19 Receiving Low Flow or No Supplemental Oxygen

**DOI:** 10.1093/ofid/ofab466.1645

**Published:** 2021-12-04

**Authors:** Eleftherios Mylonakis, Selin Somersan-Karakaya, Sumathi Sivapalasingam, Shazia Ali, Yiping Sun, Rafia Bhore, Jingning Mei, Jutta Miller, Lisa Cupelli, Andrea T Hooper, Jennifer D Hamilton, Cynthia Pan, Viet Pham, Yuming Zhao, Romana Hosain, Adnan Mahmood, John D Davis, Kenneth C Turner, Yunji Kim, Amanda Cook, Vidya Menon, Jason C Wells, Bari Kowal, Yuhwen Soo, A Thomas DiCioccio, Gregory P Geba, Neil Stahl, Leah Lipsich, Ned Braunstein, Gary Herman, George D Yancopoulos, David M Weinreich

**Affiliations:** 1 Brown University, Providence, RI; 2 Regeneron Pharmaceuticals Inc., Tarrytown, New York; 3 Regeneron Pharmaceuticals Inc, Tarrytown, New York; 4 Lincoln Medical Center, New York, New York; 5 The Oregon Clinic, Portland, Oregon

## Abstract

**Background:**

Casirivimab and imdevimab (CAS/IMDEV) is authorized for emergency use in the US for outpatients with COVID-19. We present results from patient cohorts receiving low flow or no supplemental oxygen at baseline from a phase 1/2/3, randomized, double-blinded, placebo (PBO)-controlled trial of CAS/IMDEV in hospitalized patients (pts) with COVID-19.

**Methods:**

Hospitalized COVID-19 pts were randomized 1:1:1 to 2.4 g or 8.0 g of IV CAS/IMDEV (co-administered) or PBO. Primary endpoints were time-weighted average (TWA) change in viral load from baseline (Day 1) to Day 7; proportion of pts who died or went on mechanical ventilation (MV) through Day 29. Safety was evaluated through Day 57. The study was terminated early due to low enrollment (no safety concerns).

**Results:**

Analysis was performed in pooled cohorts (low flow or no supplemental oxygen) as well as combined treatment doses (2.4 g and 8.0 g). The prespecified primary virologic analysis was in seronegative (seroneg) pts (combined dose group n=360; PBO n=160), where treatment with CAS/IMDEV led to a significant reduction in viral load from Day 1–7 (TWA change: LS mean (SE): -0.28 (0.12); 95% CI: -0.51, -0.05; P=0.0172; **Fig. 1**). The primary clinical analysis had a strong positive trend, though it did not reach statistical significance (P=0.2048), and 4/6 clinical endpoints prespecified for hypothesis testing were nominally significant (**Table 1**). In seroneg pts, there was a 47.0% relative risk reduction (RRR) in the proportion of pts who died or went on MV from Day 1–29 (10.3% treated vs 19.4% PBO; nominal P=0.0061; **Fig. 2**). There was a 55.6% (6.7% treated vs 15.0% PBO; nominal P=0.0032) and 35.9% (7.3% treated vs 11.5% PBO; nominal P=0.0178) RRR in the prespecified secondary endpoint of mortality by Day 29 in seroneg pts and the overall population, respectively (**Fig. 2**). No harm was seen in seropositive patients, and no safety events of concern were identified.

Figure 1: TWA daily viral load decreased from baseline (Day 1) in seronegative patients receiving low flow or no supplemental oxygen

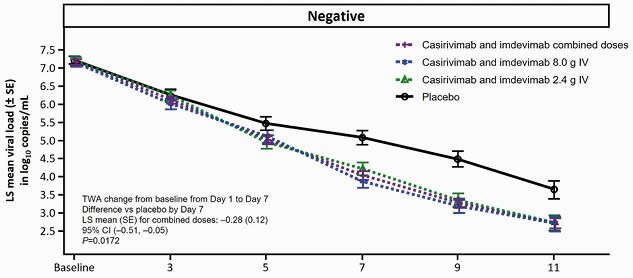

Table 1. Primary virologic and clinical endpoints

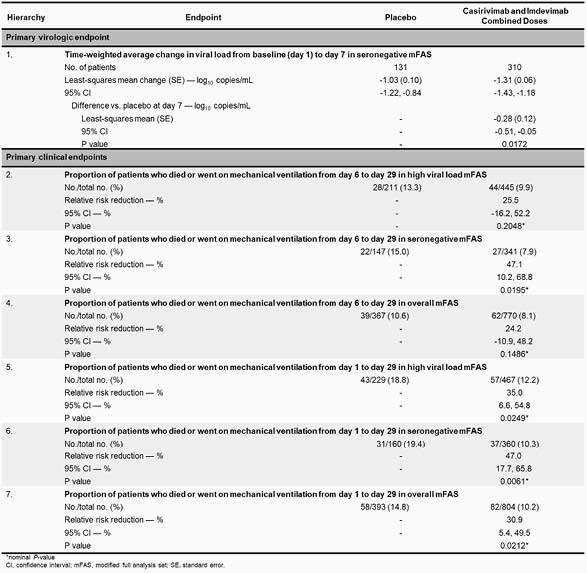

Figure 2: Clinical outcomes in hospitalized patients receiving low flow or no supplemental oxygen*

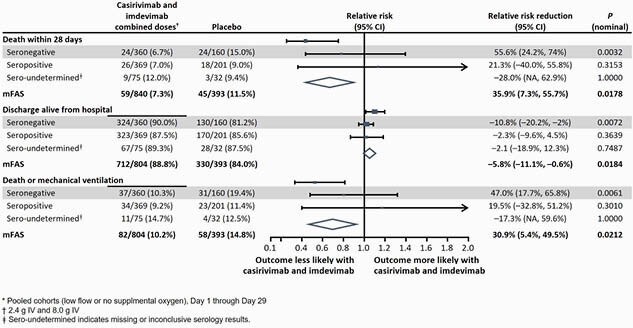

**Conclusion:**

Co-administration of CAS/IMDEV led to a significant reduction in viral load in hospitalized, seroneg pts requiring low flow or no supplemental oxygen. In seroneg pts and the overall population, treatment also demonstrated clinically meaningful, nominally significant reductions in 28-day mortality and proportion of pts dying or requiring MV.

**Disclosures:**

**Eleftherios Mylonakis, MD, PhD**, **BARDA** (Other Financial or Material Support, HHSO100201700020C)**Chemic labs/KODA therapeutics** (Grant/Research Support)**Cidara** (Grant/Research Support)**Leidos Biomedical Research Inc/NCI** (Grant/Research Support)**NIH/NIAID** (Grant/Research Support)**NIH/NIGMS** (Grant/Research Support)**Pfizer** (Grant/Research Support)**Regeneron** (Grant/Research Support)**SciClone Pharmaceuticals** (Grant/Research Support) **Selin Somersan-Karakaya, MD**, **BARDA** (Other Financial or Material Support, HHSO100201700020C)**Regeneron Pharmaceuticals, Inc.** (Employee, Shareholder) **Sumathi Sivapalasingam, MD**, **BARDA** (Other Financial or Material Support, HHSO100201700020C)**Excision BioTherapeutics** (Employee)**Regeneron Pharmaceuticals, Inc.** (Shareholder, Other Financial or Material Support, Royalties, patents planned, issued or pending, former employee) **Shazia Ali, PharmD**, **BARDA** (Other Financial or Material Support, HHSO100201700020C)**Regeneron Pharmaceuticals, Inc.** (Employee, Shareholder) **Yiping Sun, PhD**, **BARDA** (Other Financial or Material Support, HHSO100201700020C)**Regeneron Pharmaceuticals, Inc.** (Employee, Shareholder) **Rafia Bhore, PhD**, **BARDA** (Other Financial or Material Support, HHSO100201700020C)**Regeneron Pharmaceuticals, Inc.** (Employee, Shareholder) **Jingning Mei, PhD**, **BARDA** (Other Financial or Material Support, HHSO100201700020C)**Regeneron Pharmaceuticals, Inc.** (Employee, Shareholder) **Jutta Miller, BS, RN**, **BARDA** (Other Financial or Material Support, HHSO100201700020C)**Regeneron Pharmaceuticals, Inc.** (Employee, Shareholder) **Lisa Cupelli, PhD**, **BARDA** (Other Financial or Material Support, HHSO100201700020C)**Regeneron Pharmaceuticals, Inc.** (Employee) **Andrea T. Hooper, PhD**, **BARDA** (Other Financial or Material Support, HHSO100201700020C)**Pfizer, Inc.** (Shareholder, Other Financial or Material Support, Former employee)**Regeneron Pharmaceuticals, Inc.** (Employee, Shareholder, Royalties, patents planned, issued or pending) **Jennifer D. Hamilton, PhD**, **BARDA** (Other Financial or Material Support, HHSO100201700020C)**Regeneron Pharmaceuticals, Inc.** (Employee, Shareholder, Royalties, patents planned, issued or pending) **Cynthia Pan, BPharm**, **BARDA** (Other Financial or Material Support, HHSO100201700020C)**Regeneron Pharmaceuticals, Inc.** (Employee, Shareholder) **Viet Pham, BS**, **BARDA** (Other Financial or Material Support, HHSO100201700020C)**Regeneron Pharmaceuticals, Inc.** (Employee, Shareholder) **Yuming Zhao, MS**, **BARDA** (Other Financial or Material Support, HHSO100201700020C)**Regeneron Pharmaceuticals, Inc.** (Employee, Shareholder) **Romana Hosain, MD, MPH**, **BARDA** (Other Financial or Material Support, HHSO100201700020C)**Regeneron Pharmaceuticals, Inc.** (Employee, Shareholder) **Adnan Mahmood, MD**, **BARDA** (Other Financial or Material Support, HHSO100201700020C)**Regeneron Pharmaceuticals, Inc.** (Employee, Shareholder) **John D. Davis, PhD**, **BARDA** (Other Financial or Material Support, HHSO100201700020C)**Regeneron Pharmaceuticals, Inc.** (Employee, Shareholder) **Kenneth C. Turner, PhD**, **BARDA** (Other Financial or Material Support, HHSO100201700020C)**Regeneron Pharmaceuticals, Inc.** (Employee, Shareholder, Royalties, patents planned, issued or pending) **Yunji Kim, PharmD**, **BARDA** (Other Financial or Material Support, HHSO100201700020C)**Regeneron Pharmaceuticals, Inc.** (Employee, Shareholder) **Amanda Cook, BS, Dip Reg Aff**, **BARDA** (Other Financial or Material Support, HHSO100201700020C)**Regeneron Pharmaceuticals, Inc.** (Employee, Shareholder) **Jason C. Wells, MD**, **BARDA** (Other Financial or Material Support, HHSO100201700020C) **Bari Kowal, MS**, **BARDA** (Other Financial or Material Support, HHSO100201700020C)**Regeneron Pharmaceuticals, Inc.** (Employee, Shareholder) **Yuhwen Soo, PhD**, **BARDA** (Other Financial or Material Support, HHSO100201700020C)**Regeneron Pharmaceuticals, Inc.** (Employee, Shareholder) **A. Thomas DiCioccio, PhD**, **BARDA** (Other Financial or Material Support, HHSO100201700020C)**Regeneron Pharmaceuticals, Inc.** (Employee, Shareholder) **Gregory P. Geba, MD, DrPH**, **BARDA** (Other Financial or Material Support, HHSO100201700020C)**Regeneron Pharmaceuticals, Inc.** (Shareholder) **Neil Stahl, PhD**, **BARDA** (Other Financial or Material Support, HHSO100201700020C)**Regeneron Pharmaceuticals, Inc.** (Employee, Shareholder, Royalties, patents planned, issued or pending) **Leah Lipsich, PhD**, **BARDA** (Other Financial or Material Support, HHSO100201700020C)**Regeneron Pharmaceuticals, Inc.** (Employee, Shareholder) **Ned Braunstein, MD**, **BARDA** (Other Financial or Material Support, HHSO100201700020C)**Regeneron Pharmaceuticals, Inc.** (Employee, Shareholder) **Gary Herman, MD**, **BARDA** (Other Financial or Material Support, HHSO100201700020C)**Regeneron Pharmaceuticals, Inc.** (Employee, Shareholder, Royalties, patents planned, issued or pending) **George D. Yancopoulos, MD, PhD**, **BARDA** (Other Financial or Material Support, HHSO100201700020C)**Regeneron Pharmaceuticals, Inc.** (Employee, Shareholder, Royalties, patents planned, issued or pending) **David M. Weinreich, MD**, **BARDA** (Other Financial or Material Support, HHSO100201700020C)**Regeneron Pharmaceuticals, Inc.** (Employee, Shareholder)

